# Biochemical and genetic diversity of carbohydrate-fermenting and obligate amino acid-fermenting hyper-ammonia-producing bacteria from Nellore steers fed tropical forages and supplemented with casein

**DOI:** 10.1186/s12866-015-0369-9

**Published:** 2015-02-14

**Authors:** Cláudia Braga Pereira Bento, Analice Cláudia de Azevedo, Edenio Detmann, Hilário Cuquetto Mantovani

**Affiliations:** Departamento de Microbiologia, Universidade Federal de Viçosa, Viçosa, MG 36570-000 Brazil; Departamento de Zootecnia, Universidade Federal de Viçosa, Viçosa, Minas Gerais Brazil

**Keywords:** Deamination, Clostridiales, ionophores, dietary protein, PCR-DGGE

## Abstract

**Background:**

Dietary protein plays a major role in ruminant nutrition, and protein supplementation is a widespread practice among farmers in the tropics. Ruminal bacteria are the main agents of dietary protein and amino acid degradation, yet few studies have focused on the isolation and characterization of hyper-ammonia-producing bacteria in animals fed tropical diets or supplemented with rumen-degradable proteins. This work investigated the bacterial community diversity of the rumen of Nellore steers fed tropical forages, with or without casein supplementation. We also isolated and characterized ruminal bacteria showing high levels of ammonia production.

**Results:**

Polymerase chain reaction–denaturing gradient gel electrophoresis analysis indicated no differences in the ruminal bacterial community composition between the control and supplemented animals. Amino acid-fermenting bacteria (n = 250) were isolated from crossbred Nellore steers fed Tifton 85 (*Cynodon* sp.) using trypticase as the sole carbon and organic nitrogen source in the enrichment and isolation media. The deamination rates in isolates obtained from steers supplemented with casein showed a higher incidence of deamination rates >350 nmol NH_3_ mg protein^−1^ min^−1^ (*P* < 0.05), whereas isolates obtained from steers without supplementation showed deamination rates <200 nmol NH_3_ mg protein^−1^ min^−1^. Although most isolates (84%) could ferment carbohydrates, none could hydrolyze proteins or use urea to sustain growth. All isolates were sensitive to lasalocid and monensin (1 μmol l^−1^), and similarity analysis of the 16S rRNA sequences indicated a predominance of bacteria from the order Clostridiales, with variable homology (73–99%) to known bacterial species.

**Conclusions:**

These results expand what is known about the biochemical and genetic diversity of hyper-ammonia-producing bacteria, and emphasize the role of carbohydrate-fermenting bacteria in ammonia production in the rumen.

**Electronic supplementary material:**

The online version of this article (doi:10.1186/s12866-015-0369-9) contains supplementary material, which is available to authorized users.

## Background

In tropical regions, the availability of forage and the quantity and quality of protein available to ruminants varies depending on the season. Brazil, being classed as a tropical region, has two distinct seasons in terms of the availability of feedstuffs for ruminants: the dry season (April–September), and the rainy season (October–March) [1,2]. In the dry season, forages have a high insoluble fiber and lignin content; however, the crude protein content is usually <7%, which is considered limiting for the adequate function of ruminal microorganisms [1]. During the rainy season, forages show adequate levels of crude protein, but microbial protein synthesis is limited because of the high degradability of the dietary protein [2,3]. Protein supplementation is therefore a widespread practice in Brazil to provide a more balanced diet [4,5]. In the dry season, rumen-degradable protein (RDP) is provided to animals, whereas in the rainy season, rumen-undegradable protein is added to ruminant rations [4,6].

Dietary protein in the rumen is degraded by the coordinated action of proteases, peptidases, and deaminases, producing peptides, amino acids, and ammonia, respectively [7]. Most of the ammonia produced is utilized by ruminal bacteria as a source of nitrogen. However, the catabolism of carbohydrates can occur faster than the incorporation of ammonia by rumen microorganisms. Ammonia accumulated in the rumen can be absorbed through the rumen wall, and is then converted to urea in the liver and excreted in the urine [7]. Recycling of urea in the saliva can help to balance the need for nitrogen in the rumen, and previous work indicated that 10–40% of ruminal urea requirements could be supplied through urea secreted in saliva [8].

Previous reports have confirmed the role of several rumen bacteria in dietary protein deamination [9-16]. Some of these bacteria can also ferment carbohydrates in the rumen (e.g. *Butyrivibrio fibrisolvens*, *Prevotella ruminicola*, *Eubacterium ruminantium*). However, these carbohydrate-fermenting species cannot fully explain the deamination rates of mixed ruminal bacterial cultures [14,15,17]. Within the last 25 years, several Gram-positive and monensin-sensitive bacteria with high specific activity for ammonia production have been isolated from ruminants [10-16]. These hyper-ammonia-producing bacteria (HAB) were initially characterized as obligate amino-acid fermenters, and were found in small numbers in the rumen using molecular probes [18].

The physiological and genetic diversity of HAB was further explored by several different groups. McSweeney et al. [19] isolated a group of bacteria that could grow rapidly on peptides and amino acids, and had proteolytic activity, from the rumen fluid of sheep and goats fed tannin-rich diets based on *Calliandra calothyrsus*. Although these bacteria were reported as HABs, the level of ammonia and biomass produced in the absence of carbohydrates was often low. The idea that biochemical diversity within the HAB group was greater than initially thought was emphasized by the presence of significant populations of HABs in pasture-grazed New Zealand ruminants [12]. This idea was further supported by the work of Russell [14], who demonstrated that *Fusobacterium necrophorum*, a Gram-negative, nonmotile, and rod-shaped bacterium, degraded lysine with a very high rate of deamination (2,400 nmol NH_3_ mg protein^−1^ min^−1^). *F. necrophorum* was characterized as a carbohydrate-fermenting HAB that could produce acetic acid, butyric acid, and ammonia from the fermentation of lysine. Further work has indicated that HABs are also involved in protein metabolism in other mammals, and could play a major role in human health [20,21].

However, studies focusing on the isolation, characterization, and quantification of HAB in animals fed tropical diets or supplemented with RDP are lacking. The utilization of dietary protein is a major factor limiting productivity in the tropics, and protein is the most expensive component of cattle diets. Thus, understanding the role of ruminal bacteria in dietary protein metabolism is essential for developing strategies to improve the efficiency of nitrogen retention in the animal, and to lower the costs of livestock production in these countries.

This work aimed to: 1) determine if there were differences in ruminal bacterial community composition between crossbred Nellore steers fed tropical forages, with or without casein supplementation; 2) isolate ruminal bacteria involved in amino acid and peptide metabolism from the rumen of steers, with or without RDP; 3) phenotypically and biochemically characterize isolates with high specific activity for ammonia production; and 4) determine the phylogenetic relationship of these HABs.

## Results

**Figure 1:**
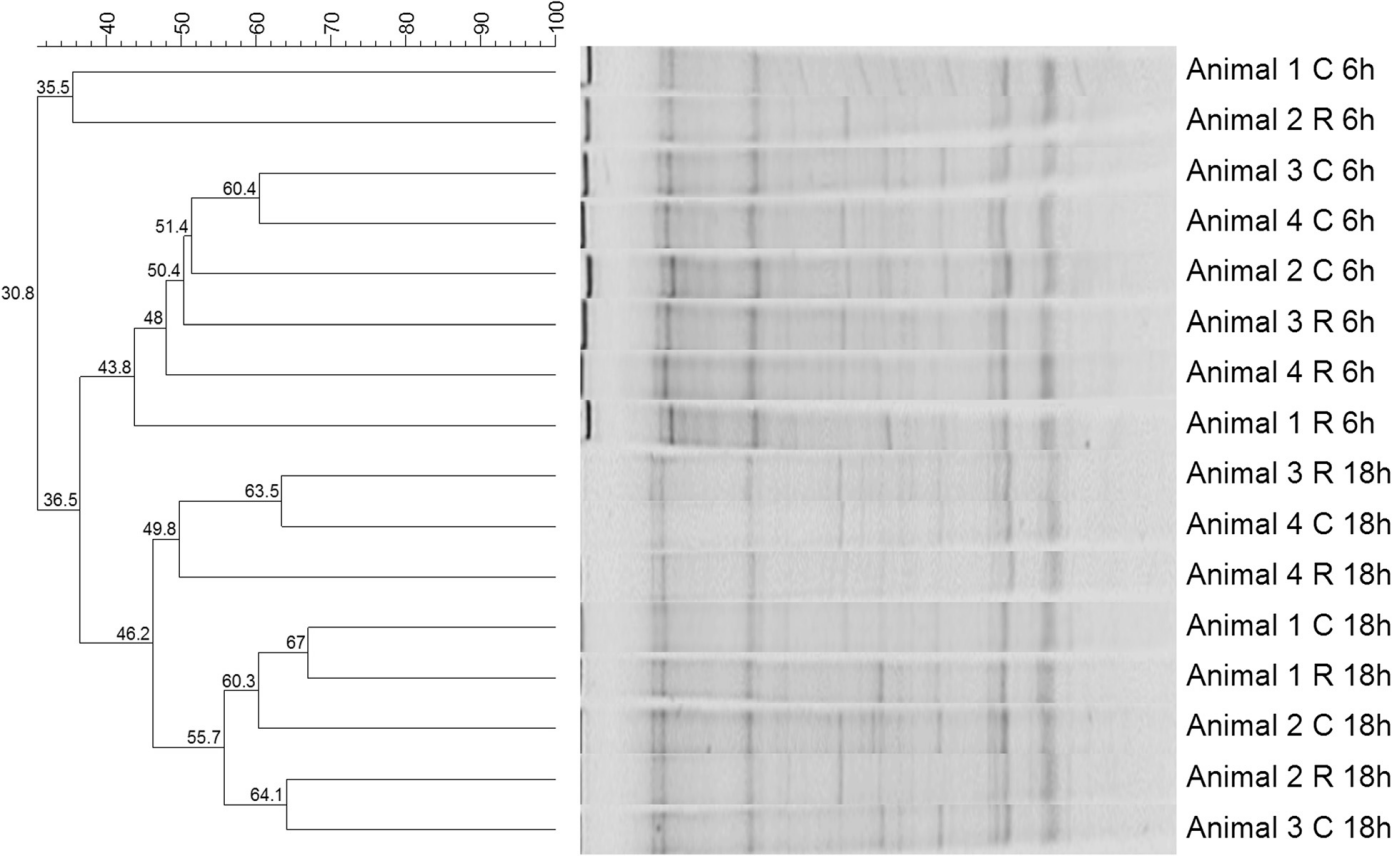
**Electrophoretic profile of 16S rRNA gene sequences of rumen bacteria obtained by denaturing gradient gel electrophoresis.** The animals were fed hay, with or without casein supplementation. The profiles were obtained after nested-PCR amplification of genomic DNA extracted from ruminal fluid. The UPGMA dendrogram was generated using BioNumerics 5.1. (C) Control animals receiving only tropical forage; (R) supplemented animals receiving daily ruminal infusion of 230 g casein; (6 h) rumen fluid collected 6 h after the morning feeding; (18 h) rumen fluid collected 6 h after the evening feeding.

In this study, we used polymerase chain reaction–denaturing gradient gel electrophoresis (PCR-DGGE) to evaluate the bacterial community composition of different animals consuming tropical forages, with or without casein supplementation infused directly into the rumen of the fistulated animals. Amplification of the 16S rRNA V3 region revealed a range of 28–35 amplicons (average of 32 amplicons) that showed low similarity between treatments (with and without casein infusion into the rumen) and between the animals used in this study. This result indicated that each animal had a different microbial community structure, and the only parameter that grouped samples together was the time of sampling (Figure 1), despite the fact that the ammonia concentration increased by 76% (*P* < 0.05) in the rumen of the animals supplemented with casein.

Because the ruminal infusion of RDP did not cause noticeable changes in the bacterial community composition, we hypothesized that the population of specialized bacteria involved in protein degradation and amino acid deamination was not being accessed by PCR-DGGE. To test this hypothesis, we decided to use culture-dependent techniques and enrichments in batch and continuous cultures to estimate the abundance, and to characterize the predominant phenotypes of amino acid-fermenting bacteria from crossbred Nellore steers fed tropical forages. A total of 250 individual isolates were isolated from fistulated animals, including 123 cultures obtained from control animals fed only basal forages, and 127 obtained from animals that received casein supplementation in the rumen.

The specific activity of deamination (SAD) and total ammonia production of each isolate was determined, and 30 isolates showing SAD ≥100 nmol NH_3_ mg protein^−1^ min^−1^ and ammonia concentration ≥25 mmol l^−1^ after 24 h of growth were selected for further characterization. All experiments were performed using trypticase as the sole nitrogen source. Morphological analysis of the cultures by light microscopy indicated that the majority of the isolates were motile (60%) Gram-positive bacilli (the only exceptions were isolates C34 and R36, which were Gram-negative), and were able to form spores (80%) (Table 1). When bacteria were grown in anaerobic mineral medium containing trypticase as the sole source of carbon and energy, the maximum optical density (600 nm) ranged from 0.406 (isolate R60) to 0.838 (isolate R23) (Table 1).

**Table 1:** Phenotypic characteristics of hyper-ammonia-producing bacterial isolates* obtained from Nellore steers

Isolate ID**	**Cell shape/gram staining**	**Enrich.** ^**1**^	**Cultural characteristics**	**Mot.** ^**2**^	**Spore**	**Maximum OD** ^**3**^	**SAD** ^**4**^	**NH**_**3**_^**5**^
C11	Rod-shaped. Gram +	15	White/small colony^6^	+	-	0.631 ± 0.025	114.85 ± 3.14	34.30 ± 1.88
C33	Rod-shaped. Gram +	15	White/large colony^8^	+	+	0.820 ± 0.091	129.46 ± 2.87	37.25 ± 3.67
C34	Rod-shaped. Gram -	15	Translucent/small colony	-	-	0.776 ± 0.052	112.69 ± 1.23	34.44 ± 1.15
C37	Rod-shaped. Gram +	15	White/small colony	+	+	0.681 ± 0.001	101.25 ± 4.57	33.29 ± 3.47
C47	Rod-shaped. Gram +	1.5	White/small colony	-	+	0.575 ± 0.037	146.47 ± 2.51	34.87 ± 0.46
C48	Rod-shaped. Gram +	1.5	White/small colony	+	+	0.647 ± 0.071	129.48 ± 2.85	41.29 ± 3.93
C51	Rod-shaped. Gram +	1.5	White/small colony	+	+	0.637 ± 0.016	106.86 ± 1.25	36.21 ± 0.88
C54	Rod-shaped. Gram +	1.5	White/medium colony^7^	-	+	0.562 ± 0.041	128.01 ± 3.98	28.35 ± 2.01
C89	Rod-shaped. Gram +	15	Translucent/medium colony	-	+	0.675 ± 0.045	203.27 ± 5.76	33.65 ± 1.83
C114	Rod-shaped. Gram +	1.5	Translucent/small colony	+	+	0.434 ± 0.039	278.25 ± 4.65	33.90 ± 0.96
C116	Rod-shaped. Gram +	15	Translucent border and black center/medium colony	-	+	0.332 ± 0.015	286.45 ± 1.32	25.15 ± 5.21
C117	Rod-shaped. Gram +	15	Translucent border and black center/medium colony	+	+	0.671 ± 0.048	186.98 ± 2.03	39.99 ± 1.69
C118	Rod-shaped. Gram +	15	Translucent border and black center/medium colony	-	+	0.609 ± 0.092	222.70 ± 3.04	34.15 ± 0.25
C122	Rod-shaped. Gram +	15	Translucent/large colony	+	-	0.647 ± 0.045	283.41 ± 3.15	35.09 ± 1.98
R15	Rod-shaped. Gram +	15	Translucent/small colony	+	+	0.601 ± 0.022	143.75 ± 2.53	39.02 ± 2.01
R21	Rod-shaped. Gram +	1.5	Yellow/medium colony	+	-	0.577 ± 0.016	469.23 ± 6.76	26.01 ± 3.62
R23	Rod-shaped. Gram +	1.5	Translucent/medium colony	+	-	0.838 ± 0.093	608.18 ± 7.31	30.88 ± 6.25
R34	Rod-shaped. Gram +	15	Translucent/small colony	+	+	0.742 ± 0.056	432.04 ± 4.08	44.17 ± 4.82
R36	Rod-shaped. Gram -	15	White/medium colony	-	-	0.561 ± 0.037	298.91 ± 2.29	33.33 ± 1.19
R40	Rod-shaped. Gram +	15	White/large colony	+	+	0.594 ± 0.048	180.92 ± 2.97	30.59 ± 2.05
R50	Rod-shaped. Gram +	1.5	Translucent/medium colony	+	+	0.660 ± 0.008	371.16 ± 4.47	38.01 ± 0.87
R51	Rod-shaped. Gram +	1.5	Translucent/small colony	-	+	0.662 ± 0.089	207.05 ± 1.64	26.55 ± 3.44
R60	Rod-shaped. Gram +	1.5	White/large colony	-	+	0.406 ± 0.032	475.22 ± 8.91	27.42 ± 7.24
R61	Rod-shaped. Gram +	1.5	Translucent/medium colony	-	+	0.435 ± 0.015	492.63 ± 6.03	27.42 ± 7.31
R63	Rod-shaped. Gram +	1.5	Translucent/large colony	+	+	0.415 ± 0.076	394.92 ± 3.87	25.47 ± 9.97
R90	Rod-shaped. Gram +	15	Translucent/large colony	+	+	0.612 ± 0.077	345.80 ± 4.69	27.31 ± 3.22
R91	Rod-shaped. Gram +	15	Yellow/large colony	+	+	0.535 ± 0.015	332.12 ± 3.36	32.50 ± 5.18
R96	Rod-shaped. Gram +	1.5	Yellow/medium colony	-	+	0.421 ± 0.045	381.73 ± 4.05	21.04 ± 6.53
R97	Rod-shaped. Gram +	1.5	Yellow/medium colony	-	+	0.573 ± 0.106	440.90 ± 6.48	21.55 ± 6.25
R107	Rod-shaped. Gram +	15	White/large colony	+	+	0.546 ± 0.080	197.60 ± 3.54	32.32 ± 1.59

*The isolates were cultured in anaerobic mineral medium supplemented with 15 g l^−1^ trypticase for up to 48 h at 39°C.

**C, isolates obtained from animals not supplemented with casein; R, isolates obtained from animals that received ruminal infusion of casein.

Symbols: (+) present; (−) absent.

^1^Concentration of trypticase (g l^−1^) used in enrichment cultures; ^2^Motility; ^3^Maximum optical density (600 nm); ^4^Specific activity of deamination (nmol NH_3_ mg protein^−1^ min^−1^); ^5^Ammonia (mmol l^−1^); ^6^small colony (≤0.5 mm); ^7^medium colony (>0.5 ≤1.0 mm); ^8^large colony (> 1.0 mm).

Values are provided as the mean ± standard deviation of the mean.

**Figure 2:**
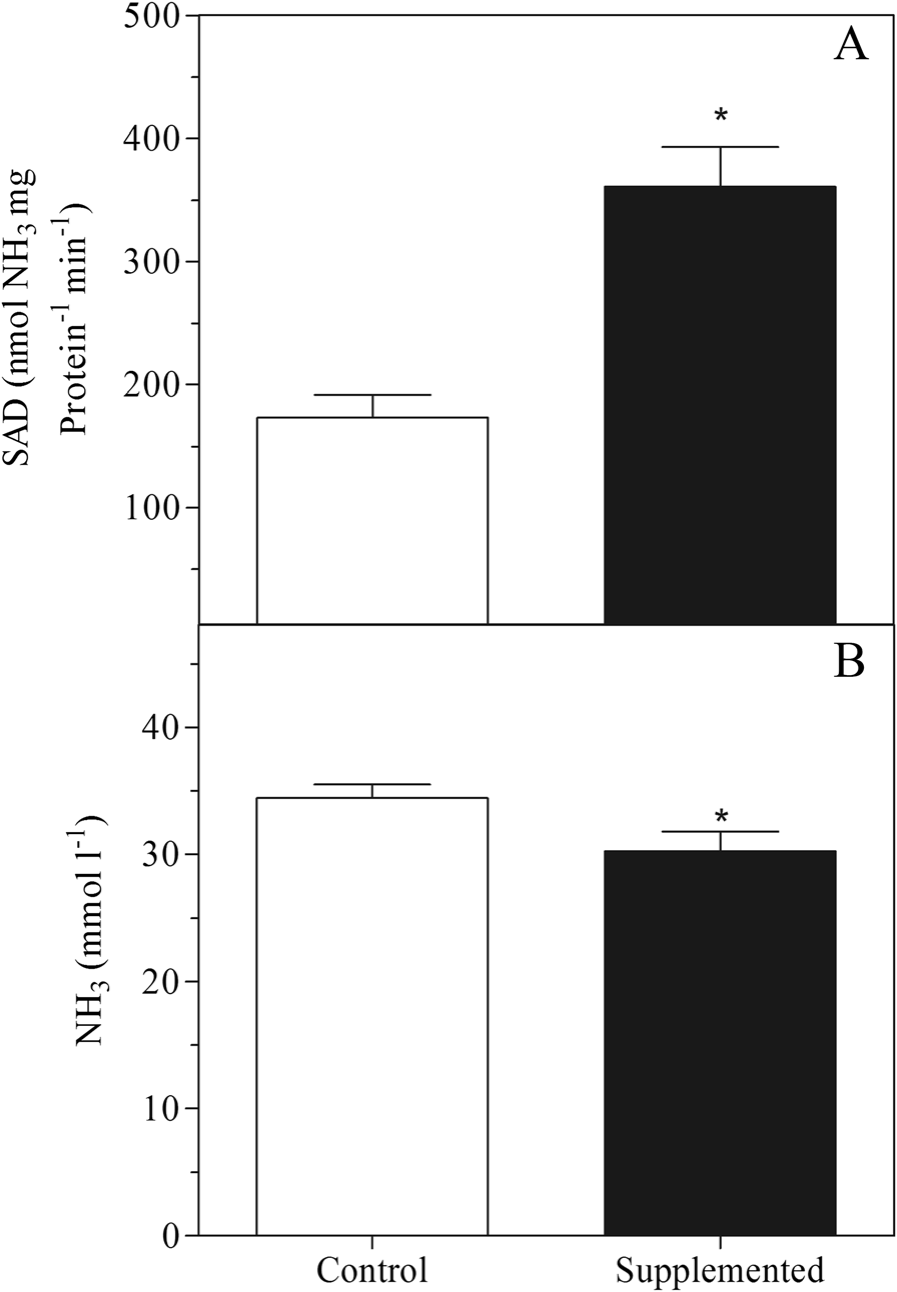
**Specific activity of deamination (A) and ammonia concentration (NH**_**3**_ **) (B) of hyper-ammonia-producing bacteria (HAB) isolated from Nellore steers.** The isolates were grown on mineral medium containing 15 g l^−1^ trypticase. Control = deamination activity of HAB isolates obtained from animals without casein supplementation (n = 14); supplemented = deamination activity of HAB isolates obtained from animals supplemented with daily infusions of casein (n = 16). (*) indicates significance at 5% probability by the Tukey test.

Mixed rumen microorganisms from steers supplemented with casein were significantly more likely to show deamination rates greater than 350 nmol NH_3_ mg protein^−1^ min^−1^ (*P* < 0.05), while mixed cultures obtained from steers without supplementation showed SAD lower than 200 nmol NH_3_ mg protein^−1^ min^−1^ (Figure 2). Among the pure cultures, isolate R23 showed the highest SAD (608 nmol NH_3_ mg protein^−1^ min^−1^), while isolate R34 produced the highest total ammonia concentration *in vitro* (44.2 mmol l^−1^) (Table 1). The average total concentration of ammonia produced by pure cultures after 24 h of incubation was 32.1 ± 5.5 mmol l^−1^ (Table 1). Isolates C116, R96, and R97 produced the lowest concentrations of ammonia, and were among the cultures showing reduced optical densities when grown in trypticase.

Analysis of fermentation products by high-performance liquid chromatography (HPLC) demonstrated that most bacteria produced a variety of short-chain fatty acids from trypticase fermentation, with a predominance of acetic acid, propionic acid, butyric acid, isobutyric acid, isovaleric acid, and formic acid (Table 2). The total concentration of volatile fatty acids in individual cultures ranged from 12.3 mmol l^−1^ (isolate R97) to 79.9 mmol l^−1^ (isolate C48) (Table 2). Three groups of bacteria, named high, medium, and low fermenters, were separated based on the concentration of fermentation products obtained from trypticase utilization. High fermenters produced, on average, 66.4 ± 7.4 mmol l^−1^ of total organic acids from 15 g l^−1^ trypticase (range: 53.7–79.9 mmol l^−1^), while the average concentration of total organic acids in the cell-free supernatants of medium and low fermenter strains was 42.5 ± 4.0 (range: 34.5–49.2 mmol l^−1^) and 22.3 ± 5.9 mmol l^−1^ (range: 12.2–30.6 mmol l^−1^), respectively. The proportion of isovaleric acid in high fermenter strains was 2.6- and 4.7-fold greater than in medium and low fermenter strains, respectively, whereas these latter two groups of bacteria had, on average, greater proportions of isobutyric or formic acid in their fermentation end-products. However, the medium and low fermenter isolates R61, R21, R60, and R63 did not produce any formic acid from amino acid fermentation. Succinic acid was also detected as a fermentation product of some HAB isolates, but the concentration was always ≤6.6 mmol l^−1^.

**Table 2:** **Fermentation products (mmol l** ^**−1**^ ) of hyper-ammonia-producing bacterial isolates* obtained from Nellore steers

**Concentration of fermentation end-products**	**Isolate ID**	**Total VFA**	**A**	**P**	**B**	**Ib**	**F**	**S**	**Iv**
		**(mmol l** ^**−1**^ **)				**(%)**			
**High**	C48	79.9 ± 2.35a	47.8	8.1	7.8	5.3	4.37	4.8	21.5
	C51	76.6 ± 1.74a	54.2	7.1	7.3	5.1	1.57	1.5	23.0
	C33	73.3 ± 3.16a	55.4	7.9	5.8	5.9	2.37	nd	22.3
	R34	71.0 ± 1.30a	37.4	3.7	5.9	5.3	1.78	1.7	44.0
	C11	68.4 ± 4.84a	69.1	11.8	6.2	7.2	1.32	nd	4.2
	R36	66.7 ± 3.14a	50.3	11.3	5.1	5.1	1.48	nd	26.5
	C47	64.4 ± 1.94a	49.2	8.4	7.7	5.8	1.95	nd	26.7
	C37	62.5 ± 0.99a	65.9	13.9	6.8	6.5	1.69	nd	5.0
	R40	60.6 ± 5.15a	37.8	10.2	5.5	4.0	9.48	nd	32.9
	R90	60.2 ± 2.34a	64.9	9.7	5.4	9.7	3.63	1.5	4.9
	R15	59.6 ± 2.90a	67.4	10.1	6.9	8.2	1.94	0.5	4.7
	R23	53.7 ± 3.47a	61.8	12.0	8.3	9.5	5.14	nd	3.0
**Medium**	C89	49.2 ± 1.69b	57.1	9.5	7.3	6.0	14.9	2.5	2.4
	C34	45.5 ± 3.25b	63.3	14.6	7.2	7.2	3.00	nd	4.5
	C122	45.0 ± 2.42b	57.9	10.4	7.3	5.6	13.44	2.9	2.2
	R50	44.2 ± 2.09b	66.8	10.1	7.3	7.8	2.89	nd	4.9
	R61	44.0 ± 3.20b	39.0	7.3	6.6	4.4	nd	nd	42.5
	C114	43.6 ± 1.05b	51.4	10.4	6.5	7.8	21.72	nd	2.0
	C118	41.6 ± 1.21b	55.7	10.1	7.4	7.9	12.63	3.2	2.6
	R51	39.0 ± 3.43b	50.1	16.9	9.0	6.8	13.43	1.6	1.9
	C54	38.3 ± 1.88b	51.5	16.5	8.8	8.3	12.86	nd	1.8
	C117	34.5 ± 3.92b	49.4	15.0	2.6	13.6	8.69	6.3	4.1
**Low**	R21	30.6 ± 6.17c	71.5	9.0	8.7	5.9	nd	nd	4.7
	R60	30.3 ± 2.56c	66.0	8.4	13.5	6.2	nd	nd	5.8
	R91	25.0 ± 1.05c	20.2	10.2	3.5	56.2	8.02	nd	1.6
	R63	22.9 ± 0.61c	64.7	11.6	10.3	7.3	nd	nd	5.9
	R107	21.5 ± 2.11c	58.8	15.4	3.6	13.5	6.59	nd	1.9
	C116	19.3 ± 4.17c	50.0	17.2	nd	15.4	10.61	6.6	nd
	R96	16.9 ± 3.34c	52.2	17.3	4.5	11.6	11.13	nd	3.0
	R97	12.2 ± 5.97c	47.8	25.7	nd	7.3	19.05	nd	nd

A = acetate; P = propionate; B = butyrate; Ib = isobutyrate; F = formate; S = succinate; Iv = isovalerate; nd = not detected.

Values are provided as the mean ± standard deviation of the mean.

*The isolates were grown in anaerobic mineral medium supplemented with 15 g l^−1^ trypticase for 24 h at 39°C.

**Averages of total volatile fatty acids followed by different letters in the same column differ at 5% probability by the Scott-Knott test.

To evaluate the ability of these isolates to ferment carbohydrates, bacteria were cultivated in medium containing pentose or hexoses as a source of carbon and energy. Most isolates (84%) could ferment glucose (2 g l^−1^), with the exception of C34, R21, R40, R50, and R51 (Table 3), while eight isolates could grow on xylose (2 g l^−1^) (Table 3). Three isolates (R21, R40, and R50) that were unable to ferment any of the sugars tested in this study were isolated from animals that received casein supplementation. Isolates C51, C89, C114, C117, and R90 could ferment all the sugars tested, but only isolate R90 was obtained from a supplemented animal (Table 3).

**Table 3:** Utilization of different carbon sources by hyper-ammonia-producing bacterial isolates* obtained from Nellore steers

Isolate ID**	**Trypticase**	**Cellobiose**	**Glucose**	**Maltose**	**Xylose**	**Casein**	**Trypticase + urea**
C11	+ + +	-	+ +	+	-	-	+
C33	+ + +	+	++	-	-	-	+
C34	+ + +	+	-	-	+	-	+
C37	+ + +	-	+ +	+	-	+	+
C47	+ + +	-	+	-	-	-	+ +
C48	+ + +	-	+ + +	+ + +	-	-	+
C51	+ + +	+	+ + +	+ + +	+	++	+ +
C54	+ + +	+ +	+	+ +	-	-	+ +
C89	+ + +	+	+ +	+ +	+	+	+ +
C114	+ +	+ +	+ +	+ +	+	+	+
C116	+ +	-	+	+	-	-	+ +
C117	+ + +	+	+	+	+	+	+ +
C118	+ + +	+ +	+	+	-	-	+ +
C122	+ + +	+	+ + +	+ +	-	+	+ +
R15	+ + +	-	+ + +	+ +	+	+	+ +
R21	+ + +	-	-	-	-	+	+
R23	+ + +	-	+	-	-	+	+ +
R34	+ + +	+	+	+ +	-	-	+
R36	+ + +	-	+ +	-	+	+	+
R40	+ + +	-	-	-	-	-	+
R50	+ + +	-	-	-	-	-	+
R51	+ + +	-	-	+	-	-	+
R60	+ +	+	+	-	-	+	+ +
R61	+ +	+	+	+	-	+	+ +
R63	+ +	+	+	+	-	+	+ +
R90	+ + +	+	+ +	+	+	+	+ +
R91	+ + +	-	+	+	-	+	+
R96	+ +	+	+ +	+	-	+	+ +
R97	+ + +	-	+	-	-	-	+
R107	+ + +	-	+	-	-	+	+ + +

*Growth using carbohydrates (2.0 g l^−1^) and trypticase (15 g l^−1^) was determined following incubation for 24 h at 39°C. Utilization of casein (4.0 g l^−1^) and trypticase (7.5 g l^−1^) plus urea (2.0 g l^−1^) was determined following incubation of the isolates at 39°C for 48 h.

**C, isolates from animals not supplemented with casein; R, isolates obtained from animals that received ruminal infusion of casein.

(−) non-growth (OD_600_ < 0.150); (+) change in OD_600_ 0.150–0.300; (+ +) change in OD_600_ 0.300–0.450; (+ + +) OD_600_ > 0.450.

Because urea supplementation is a common practice in tropical regions, we examined whether the amino acid-fermenting bacteria could also metabolize urea as a nitrogen source. Additionally, the proteolytic activity of these isolates was investigated to gain further insight into the range of substrates used by these groups of bacteria. All strains grew when cultured in basal medium containing 7.5 g l^−1^ of trypticase and 2.0 g l^−1^ of urea. However, very little ureolytic activity (mean: 3.61 ± 3.49 μmol NH_3_ mg protein^−1^ min^−1^) was observed in washed cultures incubated with 50 mmol l^−1^ urea, suggesting that this is not a suitable substrate to sustain the growth of these isolates (Table 4). Only isolates C47, C117, and R107 had ureolytic activities > 10 μmol NH_3_ mg protein^−1^ min^−1^. Additionally, little growth was observed when the isolates were cultivated with casein as the sole source of carbon and energy, and a low proteolytic activity (0.18–31.3 U μg protein^−1^) was detected in these cultures (Table 4).

**Table 4:** Proteolytic and ureolytic activities of hyper-ammonia-producing bacterial isolates* obtained from Nellore steers

Isolate ID**	**Proteolytic activity in trypticase** ^**1**^	**Proteolytic activity in Casein** ^**1**^	**Ureolytic activity in trypticase + urea** ^**2**^
C11	0.12 ± 0.01	0.86 ± 0.06	1.74 ± 0.01
C33	0.01 ± 0.01	1.18 ± 0.04	0.80 ± 0.01
C34	0.41 ± 0.01	10.87 ± 0.08	2.37 ± 0.05
C37	0.07 ± 0.01	0.40 ± 0.03	6.03 ± 0.15
C47	0.16 ± 0.01	0.89 ± 0.08	12.07 ± 0.06
C48	1.19 ± 0.03	4.30 ± 0.16	0.78 ± 0.01
C51	0.09 ± 0.01	0.18 ± 0.01	1.96 ± 0.03
C54	1.61 ± 0.01	5.17 ± 0.26	3.46 ± 0.04
C89	0.13 ± 0.01	2.52 ± 0.02	6.01 ± 0.10
C114	0.17 ± 0.01	0.66 ± 0.01	1.50 ± 0.03
C116	0.14 ± 0.01	0.83 ± 0.07	3.10 ± 0.01
C117	1.25 ± 0.01	31.26 ± 1.76	15.55 ± 0.39
C118	0.13 ± 0.01	0.91 ± 0.01	4.81 ± 0.03
C122	0.70 ± 0.01	1.36 ± 0.09	2.33 ± 0.02
R15	0.11 ± 0.01	0.38 ± 0.01	2.06 ± 0.08
R21	0.12 ± 0.01	0.81 ± 0.04	1.05 ± 0.01
R23	0.09 ± 0.01	4.02 ± 0.16	1.81 ± 0.03
R34	0.34 ± 0.02	0.91 ± 0.08	1.28 ± 0.01
R36	0.91 ± 0.01	1.55 ± 0.09	3.77 ± 0.08
R40	2.46 ± 0.02	9.48 ± 0.37	7.44 ± 0.04
R50	0.78 ± 0.03	1.65 ± 0.17	2.67 ± 0.03
R51	0.85 ± 0.01	0.87 ± 0.04	1.41 ± 0.02
R60	0.08 ± 0.01	0.61 ± 0.06	1.36 ± 0.02
R61	0.10 ± 0.01	0.42 ± 0.04	1.33 ± 0.02
R63	0.13 ± 0.01	0.46 ± 0.07	1.11 ± 0.01
R90	0.22 ± 0.01	0.79 ± 0.07	2.41 ± 0.03
R91	0.22 ± 0.01	1.38 ± 0.38	2.07 ± 0.04
R96	0.19 ± 0.01	0.68 ± 0.09	1.55 ± 0.03
R97	0.05 ± 0.01	1.17 ± 0.13	4.00 ± 0.06
R107	0.12 ± 0.01	0.99 ± 0.31	10.37 ± 0.41

*Cultures were incubated for 48 h at 39°C in anaerobic mineral medium with limited nitrogen before the enzymatic activities were determined.

**C, isolates from animals not supplemented with casein; R, isolates obtained from animals that received ruminal infusion of casein.

^1^U μg protein^−1^.

^2^μmol NH_3_ mg protein^−1^ min^−1^.

Values are given as mean ± standard deviation of the mean.

HAB species are generally sensitive to ionophores, and the addition of 1 μmol l^−1^ of lasalocid or monensin to the culture medium containing trypticase (15 g l^−1^) drastically reduced (>90%) bacterial growth and the consequent accumulation of ammonia (data not shown) of all isolates tested in this study.

The 16S rRNA genes of the 30 HAB that were selected for characterization were sequenced and compared with known sequences in the GenBank sequence database and the Ribosomal Database Project. Similarity analysis indicated that identity to the closest match in GenBank varied from 73–99%, while hierarchical taxa assignment based on Ribosomal Database Project search results returned hits similar to the GenBank at the species level. Because the sequence similarity was generally low, it was difficult to phylogenetically match isolates with previously reported bacterial species. However, sequence comparisons carried out against both databases indicated a predominance of bacteria from the order Clostridiales.

**Figure 3:**
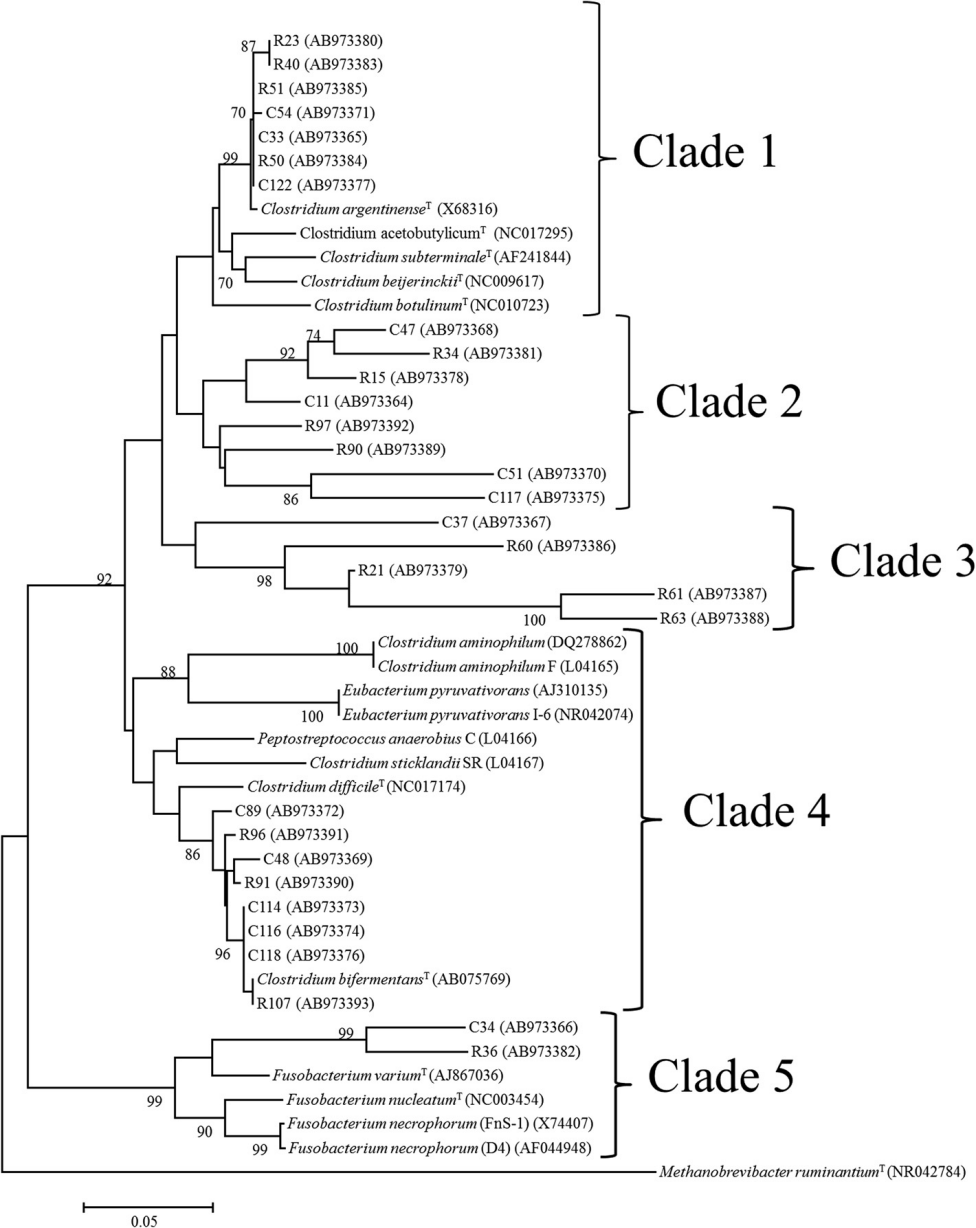
**Phylogenetic relationships of the hyper-ammonia-producing bacteria (HAB) isolated from Nellore steers.** The consensus tree, based on the neighbor*-*joining method, was constructed with 5000 repetitions using the program MEGA 4.0. Tree construction was based on 16S rRNA sequences of the HAB isolates, and included known HAB and other bacteria. The GenBank accession numbers of the bacterial strains are indicated in parentheses. Only branches with bootstrap values >70% are shown. The scale bar represents five nucleotide changes per 100 nucleotides analyzed.

Figure 3 shows the consensus phylogenetic tree obtained by bootstrap analysis (number of times the replication occurred in the group) considering 5000 replicates, and many branches were supported by bootstrap values >90%. The strains were grouped into five major clades descending from common ancestors represented as clades 1–5 in Figure 3. Seven HAB strains were most closely related to *Clostridium argentinense* (16S rRNA sequence identity 96–99%), a bacterium that is distinct from *C. botulinum*, *C. subterminale*, and *C. hastiforme* and has been isolated from soil and blood samples.

Clade 2 contained eight strains that shared a common ancestor with strains in clade 1. However, hierarchical taxon assignment within this clade was difficult as sequence analysis indicated very low homology (83–91%) between our query sequences and other 16S rRNA sequences deposited in the databases (see Additional file 1: Table S1). Five isolates that grouped in clade 3 (C37, R60, R21, R61 and R63) were also only distantly related to other species of *Clostridium* (sequence identity ≤89%).

Eight HAB isolates assigned to clade 4 were more closely related to well-known ruminal HAB (*Clostridium sticklandii* SR, *Clostridium aminophilum* F, *Peptostreptococcus anaerobius* C, and *Eubacterium pyruvativorans* I-6) that were phylogenetically grouped within Cluster XI of the Clostridiales. However, in contrast to previous studies, all isolates within clade 4 could ferment carbohydrates as their sole source of carbon and energy. In particular, isolate R107 shared 99% identity with *Clostridium bifermentans* (Additional file 1: Table S1), a bacterium that shows arginine deaminase activity and can grow with Stickland pairs of amino acids. Isolates C34 and R36 were assigned to clade 5, containing species of *Fusobacterium*, and their closest match in Genbank was *Fusobacterium varium*, although with a very low sequence similarity (84% and 91% for C34 and R36, respectively) (Additional file 1: Table S1).

## Discussion

In this work, RDP (casein) was supplemented into the rumen of Nellore steers, and changes in bacterial community composition were assessed to investigate the impact of supplementation on rumen ecology. PCR-DGGE and cluster analysis failed to discriminate the genetic diversity of the ruminal bacterial community in control (nonsupplemented) versus supplemented steers (Figure 1). Considering that species richness and population size are both components of diversity, the hypothesis that casein increases the number and/or metabolic activity of the HABs, without affecting species richness, cannot be ruled out. This idea is supported by the observation that the rate of ammonia production increased approximately 33% in the rumen fluid of supplemented steers.

Previous studies using PCR-DGGE demonstrated that grouping of treatments by diet or rumen fraction (liquid or solid) is not always possible [22,23]. Additionally, individual variation between animals has frequently been demonstrated by DGGE profiling and other molecular techniques applied for microbial community analysis, indicating that the bacterial community composition may differ substantially even between animals fed the same diet [23-26].

To identify bacteria potentially involved in protein utilization and amino acid degradation in the rumen of the steers supplemented with casein, a series of enrichments and isolations were performed in medium containing a mixture of amino acids and peptides, aimed at selecting for specialized populations of anaerobic bacteria that contribute to the metabolism of dietary nitrogen in the rumen. Our enrichment and isolation strategy was based on previous observations that carbohydrate-fermenting ruminal bacteria have deamination rates of approximately 20 nmol NH_3_ mg protein^−1^ min^−1^ [12,14], while the rates of ammonia production in obligate amino acid-fermenting bacteria are at least 10-fold greater [9,10,27].

In our study, isolates were selected as HAB if the specific activity of ammonia production was ≥100 nmol NH_3_ mg protein^−1^ min^−1^, which has been previously reported for bacteria highly active in amino acid deamination [10,12-15]. A greater deamination activity was not always related to a higher concentration of ammonia in the growth medium, suggesting that these bacterial isolates differ in their ability to tolerate ammonia accumulation.

Amino acid fermentation yielded mainly acetic, propionic, butyric, isobutyric, and isovaleric acids (Table 2), which are produced from the oxidation of carbon skeletons derived from amino acid deamination [28]. Additionally, the proportion of fermentation products varied considerably among the isolates, with high levels of acetic, propionic, isovaleric, and isobutyric acids. Specifically, isolates R34 and R61 showed fermentation yields with 44.0% and 42.6% of isovaleric acid, respectively, a fermentation product mainly related to leucine utilization (Table 2) [9]. Isobutyric acid was the main fermentation end-product (56.2%) of isolate R91, and production of this organic acid has been associated with valine fermentation [29].

Interestingly, three HAB isolates obtained in this work (R21, R40, and R50) only utilized amino acids as carbon and energy sources, a typical phenotype of the “classical HAB” [9,10], but most of the isolates with high NH_3_-producing activity could also ferment carbohydrates (Table 3). Five of these isolates (C51, C89, C114, C117, and R90) could ferment hexoses, pentoses, and disaccharides. When Eschenlauer et al. [30] screened and characterized culturable HABs obtained from the rumen of fistulated sheep, 19 anaerobic bacteria showing high rates of ammonia formation in M2 medium were isolated. However, only the nonsaccharolytic isolates showed deamination activity in medium containing trypticase as the sole source of carbon and energy. Additionally, Attwood et al. [12] previously found that HAB isolates obtained from the rumen of cattle, sheep, and deer could ferment different carbohydrates, including glucose, fructose, cellobiose, xylose, maltose, and trehalose, but the deamination rates of these isolates were not determined. A later work demonstrated that *F. necrophorum*, a ruminal HAB, could utilize glucose, maltose, and galactose, but only if a source of amino acids and peptides (such as trypticase) or yeast extract was provided in the growth medium. If these other carbon sources were not present, no growth was observed [14].

Considering that carbohydrate-fermenting HAB and obligate amino acid-fermenting HAB obtained in this study deaminated at similar rates, which has not previously been reported for other HAB, it appears that the former group might have a role in amino acid deamination not yet accounted for by previous studies. Previous estimates of the ruminal HAB population in the rumen varied from <1% to >17%, but these studies mainly used enumeration in selective media containing amino acids as the sole source of carbon and energy, or were based on mathematical models that accounted for ammonia production by specific groups of ammonia-producing ruminal bacteria [31]. Previous enumeration studies using 16S rRNA and protein profiling also indicated that populations of HAB accounted for <2% of the total ruminal bacterial population [32]. Because carbohydrate-fermenting bacteria with high rates of ammonia production were overlooked in these previous studies, their role in amino acid degradation should be assessed to fully explain nitrogen transactions in the rumen. It should also be emphasized that, under our experimental conditions, only 12% of the 250 isolates obtained from enrichment cultures had rates of deamination >100 nmol NH_3_ mg protein^−1^ min^−1^ (mean of 273.4 nmol NH_3_ mg protein^−1^ min^−1^). Nonetheless, 90% of the isolates selected as HAB could also ferment carbohydrates *in vitro*, regardless of whether they were isolated in medium containing 1.5 or 15 g l^−1^ trypticase or if they were obtained from animals with or without casein infusion into the rumen.

Most HABs isolated in this study were unable to use casein as a carbon source or grew poorly in the presence of this substrate (Table 3). Assessment of proteolytic activity showed that these isolates were not able to hydrolyze proteins to sustain growth. Similar results were observed when the isolates were grown with urea and the ureolytic activity was determined (Table 4). McSweeney et al. [19] reported a proteolytic activity of 105 U OD^−1^ for *Prevotella ruminicola* B14, while other ruminal bacteria had activities above 160 U OD^−1^. *Selenomonas ruminantium* growing in 0.3% urea produced ≥200 μmol NH_3_ mg protein^−1^ min^−1^ [33], and Dupuy et al. [34] reported a ureolytic activity of >5.6 μmol NH_3_ mg protein^−1^ min^−1^ for *Clostridium perfringens*. Interestingly, until now, most species characterized as ruminal HAB showed little or no ureolytic activity [11-14]. In a previous work, Flythe and Andries [15] isolated HABs from Boer goats that were closely related to *Peptoniphilus indolicus* and had weak ureolytic activity (<100 nmol mg cell protein^−1^ min^−1^). Ureolytic bacteria could play an important role in ruminal nitrogen metabolism by recycling urea via saliva or the rumen wall, making the amino nitrogen available for the growth of rumen microbes [8,35].

Several bacterial species described as HAB (*C. sticklandii* SR, *P. anaerobius* C, and *F. necrophorum*) are sensitive to ionophores [11,12,14,27]. Ionophores modify the movement of ions through the cell membrane, altering proton gradients and energy transduction. In an attempt to maintain ion homeostasis, bacterial cells hydrolyze ATP, and this expenditure of energy may reduce biomass formation or cause loss of viability [27]. All bacteria obtained in this study were sensitive to the ionophores monensin and lasalocid, even isolates C34 and R36 that were characterized as Gram-negative. This observation is consistent with previous data from Russell [14], who reported that *Fusobacterium* could not grow when incubated with 5 μM monensin. These results reinforce the idea that ruminal bacteria with high SAD are generally sensitive to ionophores and support previous observations that the presence of an outer membrane alone does not necessarily confer a high degree of ionophore resistance [36].

16S rRNA sequencing analysis indicated a predominance of members of the order Clostridiales among the bacteria isolated in this study. Many isolates were only distantly related to previously reported HABs or other known bacterial species (Figure 3), and the phenotypic and biochemical characterization of the isolates set them apart from other well-known HABs. Phylogenetic analysis separated these isolates into five clades, but phenotypic and genotypic diversity was observed within each group. Most isolates were Gram-positive, anaerobic, spore-forming bacteria that could grow on carbohydrates and peptides. Only isolates C34 and R36 (Figure 3, clade 5) were Gram-negative. These nonmotile rods could ferment amino acids, hexoses, and xylose, and showed low proteolytic activity. Some of these morphological and biochemical traits are consistent with those given for species of the genus *Fusobacterium*, but many other physiological characteristics are not shared with other species belonging to this genus.

These results expand our existing knowledge on the biochemical and genetic diversity of HAB, and emphasize the role of carbohydrate-fermenting bacteria in ammonia production in the rumen. To our knowledge, this is the first description of HAB isolated from *Bos indicus*, and further studies should investigate the predominance of ruminal HABs across different diets and supplementation schemes.

## Conclusions

Improving the utilization of dietary nitrogen in the rumen can have a major economic impact on ruminant nutrition. Several proposed models of rumen fermentation recognize that ruminal bacteria have different patterns of nitrogen utilization, and that HAB appear to account for a large fraction of the amino nitrogen that is converted to ammonia in the rumen, even though they are not highly abundant. Although most HAB have been described as obligate amino acid-fermenting bacteria, the isolates obtained from Nellore steers fed tropical forages in this study were also able to metabolize sugars as a carbon source, which is a competitive advantage for niche exploitation and colonization in the rumen. Additionally, the biochemical and genetic analysis of these isolates indicated several features that are distinct from previously described strains. Considering that HABs play a role in the rapid turnover of peptides and amino acids that enter the rumen, and that an optimal balance between protein degradation and microbial protein synthesis in cattle can increase milk production and reduce nitrogen excretion into the environment, strategies to target the HABs (e.g. ionophores, bacteriocins, essential oils) might have an amino acid-sparing effect in the rumen.

## Methods

### Animals, diets, and treatments

A group of four crossbred Nellore steers fistulated in the rumen with an average initial weight of 280 ± 10 kg were used in this study, in accordance with a protocol approved by the Universidade Federal de Viçosa Ethics and Animal Care and Use Committee. The animals were housed in individual stalls with water and minerals *ad libitum*. The experiment was conducted during four experimental periods, each with a duration of 29 days. Animals in different treatment groups in each experimental period were rotated to avoid residual effects of treatments. The animals were allowed to adapt to the diet for 14 days prior to sampling. The diet comprised Tifton 85 (*Cynodon* sp.) hay with an average crude protein content of 7.8–9.8%, based on dry matter. Daily feeding occurred at 6:00 a.m. and 6:00 p.m., and ruminal supplementation with casein started on the sixth day of each experimental period.

Protein supplementation aimed to provide 55% of the calculated RDP requirements for the animals (equivalent to 230 g of casein per day per animal). Supplemented animals were infused with two portions of 115 g of pure casein (Labsynth, Diadema, Brazil) directly into the rumen at feeding. Control animals received a diet of basal forage only.

### Analysis of the bacterial community composition by PCR-DGGE

To assess the genetic diversity of the ruminal microbial community, samples (50 ml) of ruminal fluid were collected 6 h after daily feeding. The samples were stored at −80°C and processed separately by treatment, time, and animal. For DNA extraction, the samples were defrosted at room temperature and processed according to the methods described by Stevenson and Weimer [37]. The first PCR reaction used the bacterial universal primers 9bfm (5′-GAGTTTGATYHTGGCTCAG-3′) and 1512uR (5′-ACGGHTACCTTGTTACGACTT-3′) to amplify the 16S rRNA genes from the genomic DNA of bacteria in rumen fluid samples [38]. The amplification reaction contained GoTaq Reaction Buffer (0.5 X), MgCl_2_ (0.5 mmol l^−1^), dNTPs (0.2 mmol l^−1^), forward primer (0.12 mmol l^−1^), reverse primer (0.12 mmol l^−1^), *Taq* DNA polymerase (0.1 u μl^−1^) (Promega Corporation, Madison, WI, USA), BSA (0.08 mg ml^−1^) and genomic DNA (0.8 ng μl^−1^). The PCR was performed with an initial denaturation at 96°C for 4 min, followed by 35 cycles of 96°C for 1 min, 56°C for 1 min, and 72°C for 2 min, followed by a final extension at 72°C for 5 min [38].

To increase the specificity of the analysis, nested-PCR was performed to amplify a shorter region (expected size of 177 bp) of the bacterial 16S rRNA gene [39,40] using the primers 341f-GC (5′-CCTACGGGAGGCAGCAGCGCCCGCCGCGCGCGGCGGGCGGGGCGGGGGCACGGGGGG-3′) and 518r (5′-ATTACCGCGGCTGCTGG-3′) [38]. The amplification reaction contained GoTaq Reaction Buffer (0.5 X), MgCl_2_ (0.5 mmol l^−1^), dNTPs (0.2 mmol l^−1^), forward primer (0.12 mmol l^−1^), reverse primer (0.12 mmol l^−1^), *Taq* DNA polymerase (0.1 u μl^−1^) (Promega Corporation, Madison, WI, USA) and BSA (0.08 mg ml^−1^). One microliter of the amplification product from the first reaction was used as the DNA template. The nested-PCR was performed in a Biocycler MG96G thermocycler with an initial denaturation of 96°C for 4 min, followed by 35 cycles of 96°C for 1 min, 56°C for 1 min, and 72°C for 30 s, followed by a final extension at 72°C for 5 min [38].

DGGE was performed in a DGGE-2401 apparatus (CBS Scientific Company, San Diego, CA, USA) using 8 μl of the PCR products from the nested-PCR and 8 μl of sample buffer (0.05% bromophenol blue, 0.05% xylene cyanol, 70% glycerol and 1× TAE (40 mmol l^−1^ Tris, 20 mmol l^−1^ acetic acid, and 1 mmol l^−1^ EDTA)). The PCR products were loaded into wells in a 8% (w/v) vertical polyacrylamide gel (acrylamide:N,N'-methylenebisacrylamide, 37.5:1) with a linear gradient of 40–60% urea/formamide.

The denaturing gradient was obtained by mixing two solutions (A and B) dispensed by an MPP-100-220 peristaltic mini-pump (CBS Scientific Company, San Diego, CA, USA). Solution A contained 100% of the denaturing agents (7 mol l^−1^ urea and 40% deionized formamide (v/v)) in 8% acrylamide: N,N'-methylenebisacrylamide (37.5:1), and solution B was prepared as for solution A but without the denaturing agents. Solutions A and B also contained ammonium persulfate (3.1 mmol l^−1^) of polymerizer and N,N,N',N'-tetramethylethylenediamine (0.0037 mmol l^−1^) catalyst. The denaturing gradient was monitored using 20 μl of the visualization dye (bromophenol blue 0.5%, xylene cyanol 0.5%, and 1× TAE). The gels were allowed to polymerize for 3 h prior to loading the DNA samples.

A mixture of 16S rRNA amplicons obtained from the genomic DNA of *Escherichia coli* ATCC 29214 (γ-Proteobacteria), *Salmonella enterica* Typhimurium ATCC 14028 (γ-Proteobacteria), *Bacillus cereus* ATCC 14579 (Firmicutes), and *Lactococcus lactis* ATCC 19435 (Firmicutes) were used as markers for bacterial species in wells located in the sides of the gel. Electrophoresis was performed at 60°C in 1× TAE at constant voltage of 150 V for 10 h. The gel was stained for 20 minutes with SYBR Gold (Invitrogen) according to manufacturer's recommendations. The gel was visualized and photo-documented using Eagle Eye (Stratagene). Gel bands were analyzed using Bionumerics 5.1 (Applied Maths, Kortrijk, Belgium). Dice’s similarity coefficient (D_sc_) was used to compare the data sets with an optimization of 1% and a tolerance of 1.5%. Clustering was performed using the unweighted pair group method (UPGMA).

### Isolation of HAB

Rumen fluid was sampled after a period of diet adaptation (14 days) and 3 h after the morning feeding. Rumen contents were obtained from control and supplemented animals, filtered through four layers of cheesecloth, and stored in stoppered Erlenmeyer flasks in insulated containers. Ruminal fluid was collected from the center of the flask and used for bacterial enrichment in mineral media. Enrichments of amino acid-fermenting bacteria were carried out at 39°C in batch or continuous cultures with anaerobic mineral (AM) medium limited in nitrogen. The AM medium contained (per liter): 292 mg K_2_HPO_4_; 240 mg KH_2_PO_4_; 480 mg Na_2_SO_4_; 480 mg NaCl; 100 mg MgSO_4_.7H_2_O; 64 mg CaCl_2_.2H_2_O; 4000 mg Na_2_CO_3_; 600 mg cysteine hydrochloride, with added vitamins and minerals [10]. Trypticase (1.5 g l^−1^ or 15 g l^−1^) was used as the only source of carbon and energy to sustain bacterial growth in batch or continuous cultures. The culture medium was prepared under a CO_2_ atmosphere free of oxygen, and the pH was adjusted to 6.5 with NaOH.

The continuous culture was inoculated with 10% (v/v) rumen fluid, using a dilution rate of 0.07 h^−1^. The optical density (600 nm) of the fermentation vessel was monitored to determine when the steady state was achieved. Enrichments using batch cultures were performed by daily transfers of 10% (v/v) inoculum into fresh AM medium containing 1.5 g l^−1^ or 15 g l^−1^ trypticase. Ammonia production was monitored at the end of each transfer (24 h incubation) until it reached a plateau (six transfers) to determine when the microbial community had stabilized.

Samples (2 ml) were collected from batch and continuous cultures and serial dilutions (10-fold increments) were performed. Aliquots (10 μl) of the dilutions were then spread onto the surface of solid AM medium containing 1.5 g l^−1^ or 15 g l^−1^ trypticase, with or without supplementation with clarified rumen fluid (30%) (a total of four different selective media). The plates were incubated at 39°C in an anaerobic chamber (Coy Laboratory Products, Gras Lake, MI, USA) for up to 72 h.

Resultant colonies showing phenotypic and morphological differences were selected and transferred to fresh liquid media corresponding to the respective isolation media. The batch cultures were incubated overnight (39°C) and were stored at −20°C [41] for subsequent phenotypic and genotypic characterization. Phenotypic characterization was performed in AM medium containing 15 g l^−1^ of trypticase. Aliquots (1 ml) obtained from actively growing cultures were used to prepare slides for differentiating morphological characteristics (form, arrangement, sporulation, motility) and Gram staining.

### Determination of SAD and ammonia concentration

Ammonia concentration was determined by the colorimetric method of Chaney and Marbach [42]. Absorbance was measured at 630 nm in a Spectronic 20D spectrophotometer (Thermo Fisher Scientific, Madison, WI, USA) and ammonium chloride (NH_4_Cl) was used as a standard. Total ammonia (mmol l^−1^) was expressed as the difference between ammonia concentration at the time of inoculation and that following 24 h of incubation. Specific deamination activity was calculated as the difference in ammonia concentration (mmol l^−1^) between time points 0 and 6 h, divided by microbial protein concentration (mg l^−1^) and the incubation time (minutes). Microbial protein was determined according to Bradford [43], using lysozyme as the standard.

### Determination of organic acid production

Organic acid production was determined by HPLC in a Dionex Ultimate 3000 dual detector HPLC apparatus (Dionex Corporation, Sunnyvale, CA, USA) coupled to a refractive index Shodex RI-101 maintained at 40°C. A Phenomenex Rezex ROA ion exchange column (300 × 7.8 mm) was used (Phenomenex Inc. Torrance, CA, USA) and was maintained at 45°C. The mobile phase contained 5 mmol l^−1^ H_2_SO_4_ and 0.35 mmol l^−1^ sodium-free EDTA, and the flow was 0.7 ml min^−1^. Rumen fluid samples (2.0 ml) were centrifuged (12,000 × *g*, 10 min) and the cell-free supernatants were treated as described by Siegfried et al. [44].

The following organic acids were used to calibrate the standard curve: acetic, succinic, formic, propionic, valeric, isovaleric, isobutyric, and butyric acids. All acids were prepared to a final concentration of 10 mmol l^−1^, except isovaleric acid (5 mmol l^−1^) and acetic acid (20 mmol l^−1^).

### Substrate utilization and enzymatic activity

To evaluate the ability of the bacterial strains to ferment carbohydrates, cultures were inoculated (1%, v/v) into basal medium containing (per liter): 0.292 g K_2_HPO_4_, 0.292 g KH_2_PO_4_, 0.48 g (NH_4_)_2_SO_4_, 0.48 g NaCl, 0.1 g MgSO_4_.7H_2_O, 0.064 g CaCl_2_.2H_2_O, 0.5 g cysteine hydrochloride, 4 g Na_2_CO_3_, 0.1 g trypticase, and 0.5 g yeast extract. The following carbon sources were tested at 2 g l^−1^: glucose, cellobiose, maltose, and xylose. Growth was determined by measuring optical density at 600 nm in a Spectronic spectrophotometer 20D (Thermo Fisher Scientific, Madison, WI, USA) following 24 h of incubation. AM medium limited in nitrogen and supplemented with 15 g l^−1^ of trypticase was used for comparison.

Proteolytic activity was determined in medium containing trypticase or casein. The strains were cultured in AM medium limited in nitrogen and supplemented with 15 g l^−1^ trypticase or 4.0 g l^−1^ casein. Samples (2 ml) were collected after 48 h of incubation to determine proteolytic activity and microbial growth (OD 600 nm). Assays for proteolytic activity were based on the method described by Forsythe [45], using 2% (w/v) azocasein as a substrate. The proteolytic activity was expressed as activity units (U) per μg protein^−1^, in which a change of 0.01 absorbance units corresponded to 1 U divided by the concentration (μg) of microbial protein.

The urease activity was determined by culturing the strains in AM medium supplemented with 7.5 g l^−1^ trypticase and 2.0 g l^−1^ urea. Samples (2 ml) were collected after 48 hours of incubation to determine the ureolytic activity and microbial protein concentration. The ureolytic activity assay followed the methods described by Cook [46]. The proteolytic activity was expressed as μmol NH_3_ mg protein^−1^ min^−1^.

To determine the sensitivity of the bacterial isolates to ionophores, cultures were grown in AM medium limited in nitrogen and supplemented with 15 g l^−1^ of trypticase. Monensin or lasalocid (1 μmol l^−1^) were added to the growth medium prior to inoculation. The OD at 600 nm was measured after 24 h of incubation and compared with control cultures without inhibitors.

### Phylogenetic analysis

Taxonomic analysis of the bacterial strains used in this study was based on 16S rRNA gene sequences. Approximately 50 ng of genomic DNA were used in the amplification reaction containing 5.0 μl GoTaq Reaction Buffer (5×), 1.0 μl dNTPs (2.5 mmol l^−1^), 1.0 μl of each primer (10 mmol l^−1^), 0.2 μl *Taq* DNA polymerase (Promega), and 2.5 μl of MgCl_2_. The universal primers 27F (5′-AGAGTTTGATCMTGG-3′) and 1392R (5′-ACGGGCGGTGTGTRC-3′) were used for the amplification reaction. PCR was performed at 94°C for 3 min followed by 30 cycles of 94°C for 1 min, 50°C for 1 min, and 72°C for 2 min, with a final extension at 72°C for 7 min. Products of each amplification reaction were analyzed by gel electrophoresis (1.5% (w/v) agarose).

The PCR products (expected size of 1365 bp) were purified and sequenced (Macrogen, Korea). The 16S rRNA sequence from each isolate was initially analyzed using the Basic Local Alignment Search Tool (BLAST). Sequences were compared with 16S ribosomal RNA sequences of bacteria and archaea deposited in the GenBank sequence database and the Ribosomal Database Project. ClustalW [47] alignments and manual gap corrections were made using the software MEGA 4.0 [48]. Phylogenetic inferences were obtained using MEGA 4.0, and the neighbor-joining method was used for tree construction [48].

### Nucleotide sequence accession numbers

The 16S rRNA gene sequences of the bacterial isolates used in this study have been deposited in the DDBJ/EMBL/GenBank databases under the accession numbers from AB973364 to AB973393. These sequences were used to construct the phylogenetic tree as well as other 16S rRNA sequences obtained in the GenBank database for the following bacterial species (corresponding accession numbers are given in parenthesis): *Clostridium argentinense* (X68316), *Clostridium acetobutylicum* (NC017295), *Clostridium subterminale* (AF241844) *Clostridium beijerinckii* (NC009617), *Clostridium botulinum*^T^ (NC010723), *Clostridium aminophilum* (DQ278862), *Clostridium aminophilum* F (L04165), *Eubacterium pyruvativorans* (AJ310135), *Eubacterium pyruvativorans* I-6 (NR042074), *Peptostreptococcus anaerobius* C (L04166), *Clostridium sticklandii* SR (L04167), *Clostridium difficile*^T^ (NC017174), *Clostridium bifermentans*^T^ (AB075769), *Fusobacterium varium*^*T*^ (AJ867036)*, Fusobacterium nucleatum*^T^ (NC003454), *Fusobacterium necrophorum* (FnS-1) (X74407), *Fusobacterium necrophorum* (D4) (AF044948) and *Methanobrevibacter ruminantium*^T^ (NR042784).

## Additional file

Additional file 1: Table S1. Sequence similarity searching results for comparison of the 16S ribosomal RNA sequences from hyper-ammonia-producing bacterial isolates obtained from Nellore steers with the 16S rRNA sequences of Bacteria and Archaea in the GenBank sequence database. Only the match sequences with best scores for each individual isolate are listed.

## Abbreviations

AM: Anaerobic mineral media
CP: Crude protein
DGGE: Denaturing gradient gel electrophoresis
NCBI: National Center for Biotechnology Information
HAB: Hyper-ammonia-producing bacteria
HPLC: High performance liquid chromatography
OD: Optical density
PCR: Polymerase chain reaction
RDP: Rumen-degradable protein
SAD: Specific activity of deamination
