# Supplementary material for: Biochemical and genetic diversity of carbohydrate-fermenting and obligate amino acid-fermenting hyper-ammonia-producing bacteria from Nellore steers fed tropical forages and supplemented with casein
Source: BMC Microbiol. 2015 Feb 14;15:28. doi: 10.1186/s12866-015-0369-9 (PMC4332921; doi:10.1186/s12866-015-0369-9)
Supplement: Additional file 1: Table S1. — Sequence similarity searching results for comparison of the 16S ribosomal RNA sequences from hyper-ammonia-producing bacterial isolates obtained from Nellore steers with the 16S rRNA sequences of Bacteria and Archaea in the GenBank sequence database. Only the match sequences with best scores for each individual isolate are listed. [file 12866_2015_369_MOESM1_ESM.docx]

**Additional file 1 Sequence similarity searching results for comparison of the 16S ribosomal RNA sequences from hyper-ammonia-producing bacterial isolates obtained from Nellore steers with the 16S rRNA sequences of Bacteria and Archaea in the GenBank sequence database. Only the match sequences with best scores for each individual isolate are listed.**

| **Sequence ID (GenBank)** | **Length** | **Query Cover (%)** | **E(17,764*)** | **% ID** |
| --- | --- | --- | --- | --- |
| **AB973364 Strain C11, 1110 nt, best scores**  *Clostridium* *subterminale* NCIMB 10746 (NR118999)  *Clostridium argentinense* ATCC 27322 (NR029232)  *Clostridium schirmacherense* AP15 (NR042448) | 955  953  952 | 95  95  95 | 0.0  0.0  0.0 | 85  85  85 |
| **AB973365 Strain C33, 1293 nt, best scores**  *Clostridium argentinense* ATCC 27322 (NR029232)  *Clostridium schirmacherense* AP15 (NR042448)  *Clostridium* *subterminale* NCIMB 10746 (NR118999) | 1206  1202  1201 | 96  96  96 | 0.0  0.0  0.0 | 96  96  96 |
| **AB973366 Strain C34, 545 nt, best scores**  *Fusobacterium* *varium* JCM 6320 (NR113384)  *Fusobacterium ulcerans* NCTC 12111 (NR044820)  *Clostridium rectum* NCIMB 10651 (NR119089) | 414  408  400 | 89  89  89 | 2e-132  2e-124  2e-107 | 84  83  81 |
| **AB973367 Strain C37, 1126 nt, best scores**  *Terrisporobacter* *mayombei* SFC-5 (NR104744)  *Terrisporobacter* *glycolicus* DSM 1288 (NR119074)  *Intestinibacter bartlettii* WAL 16138 (NR027573) | 1009  996  986 | 99  97  97 | 0.0  0.0  0.0 | 88  88  87 |
| **AB973368 Strain C47, 1089 nt, best scores**  *Terrisporobacter* *mayombei* SFC-5 (NR104744)  *Terrisporobacter* *glycolicus* DSM 1288 (NR119074)  *Asaccharospora irregularis* DSM 2635 (NR119034) | 937  938  927 | 98  98  98 | 0.0  0.0  0.0 | 83  83  82 |
| **AB973369 Strain C48, 1132 nt, best scores**  *Clostridium* *bifermentans* JCM 1386 (NR113323)  *Clostridium* *bifermentans* ATCC 638 (NR119066)  *Clostridium* *sordellii* JCM 3814 (NR113140) | 1066  1055  1043 | 97  97  96 | 0.0  0.0  0.0 | 94  93  93 |
| **AB973370 Strain C51, 408 nt, best scores**  *Clostridium argentinense* ATCC 27322 (NR029232)  *Clostridium subterminale* NCIMB 10746 (NR118999)  *Clostridium schirmacherense* AP15 (NR042448) | 322  321  321 | 88  88  88 | 5e-124  6e-123  2e-122 | 89  89  89 |
| **AB973371 Strain C54, 1328 nt, best scores**  *Clostridium argentinense* ATCC 27322 (NR029232)  *Clostridium schirmacherense* AP15 (NR042448)  *Clostridium subterminale* NCIMB 10746 (NR118999) | 1225  1221  1220 | 95  95  95 | 0.0  0.0  0.0 | 96  96  95 |
| **AB973372 Strain C89, 1195 nt, best scores**  *Clostridium bifermentans* JCM 1386 (NR113323)  *Clostridium bifermentans* ATCC 638 (NR119066)  *Clostridium ghonii* JCM 1400 (NR104696) | 1089  1078  1070 | 92  92  92 | 0.0  0.0  0.0 | 96  95  95 |
| **AB973373 Strain C114, 1237 nt, best scores**  *Clostridium* *bifermentans* JCM 1386 (NR113323)  *Clostridium* *bifermentans* ATCC 638 (NR119066)  *Clostridium* *sordellii* JCM 3814 (NR113140) | 1201  1189  1175 | 100  100  100 | 0.0  0.0  0.0 | 97  96  95 |
| **AB973374 Strain C116, 1263 nt, best scores**  *Clostridium* *bifermentans* JCM 1386 (NR113323)  *Clostridium* *bifermentans* ATCC 638 (NR119066)  *Clostridium* *sordellii* JCM 3814 (NR113140) | 1210  1198  1186 | 99  99  99 | 0.0  0.0  0.0 | 96  95  94 |
| **AB973375 Strain C117, 545 nt, best scores**  *Clostridium argentinense* ATCC 27322 (NR029232)  *Clostridium subterminale* NCIMB 10746 (NR118999)  *Clostridium schirmacherense* AP15 (NR042448) | 460  459  459 | 100  100  100 | 3e-136  1e-134  1e-134 | 83  83  83 |
| **AB973376 Strain C118, 1338 nt, best scores**  *Clostridium* *bifermentans* JCM 1386 (NR113323)  *Clostridium* *bifermentans* ATCC 638 (NR119066)  *Clostridium* *sordellii* JCM 3814 (NR113140) | 1246  1253  1220 | 95  97  95 | 0.0  0.0  0.0 | 97  95  95 |
| **AB973377 Strain C122, 1323 nt, best scores**  *Clostridium argentinense* ATCC 27322 (NR029232)  *Clostridium subterminale* NCIMB 10746 (NR118999)  *Clostridium schirmacherense* AP15 (NR042448) | 1128  1123  1122 | 90  90  90 | 0.0  0.0  0.0 | 94  94  94 |
| **AB973378 Strain R15, 1040 nt, best scores**  *Clostridium subterminale* NCIMB 10746 (NR118999)  *Clostridium argentinense* ATCC 27322 (NR029232)  *Clostridium schirmacherense* AP15 (NR042448) | 869  867  864 | 100  100  100 | 0.0  0.0  0.0 | 83  83  82 |
| **AB973379 Strain R21, 1087 nt, best scores**  *Clostridium argentinense* ATCC 27322 (NR029232)  *Clostridium subterminale* NCIMB 10746 (NR118999)  *Clostridium schirmacherense* AP15 (NR042448) | 929  926  925 | 96  96  96 | 0.0  0.0  0.0 | 86  86  86 |
| **AB973380 Strain R23, 1371 nt, best scores**  *Clostridium argentinense* ATCC 27322 (NR029232)  *Clostridium schirmacherense* AP15 (NR042448)  *Clostridium subterminale* NCIMB 10746 (NR118999) | 1171  1166  1166 | 89  89  89 | 0.0  0.0  0.0 | 96  95  95 |
| **AB973381 Strain R34, 535 nt, best scores**  *Terrisporobacter* *glycolicus* DSM 1288 (NR119074)  *Terrisporobacter* *mayombei* SFC-5 (NR104744)  *Asaccharospora irregularis* DSM 2635 (NR119034) | 465  465  466 | 99  99  99 | 2e-162  8e-162  1e-160 | 86  86  86 |
| **AB973382 Strain R36, 597 nt, best scores**  *Fusobacterium* *varium* JCM 6320 (NR113384)  *Fusobacterium ulcerans* NCTC 12111 (NR044820)  *Fusobacterium mortiferum* DSM 19809 (NR117734) | 538  531  523 | 99  99  99 | 0.0  0.0  0.0 | 91  90  88 |
| **AB973383 Strain R40, 1232 nt, best scores**  *Clostridium argentinense* ATCC 27322 (NR029232)  *Clostridium schirmacherense* AP15 (NR042448)  *Clostridium subterminale* NCIMB 10746 (NR118999) | 1211  1206  1198 | 100  100  99 | 0.0  0.0  0.0 | 98  98  98 |
| **AB973384 Strain R50 1269 nt, best scores**  *Clostridium argentinense* ATCC 27322 (NR029232)  *Clostridium schirmacherense* AP15 (NR042448)  *Clostridium subterminale* NCIMB 10746 (NR118999) | 1213  1210  1209 | 96  97  97 | 0.0  0.0  0.0 | 99  98  98 |
| **AB973385 Strain R51 1218 nt, best scores**  *Clostridium argentinense* ATCC 27322 (NR029232)  *Clostridium schirmacherense* AP15 (NR042448)  *Clostridium subterminale* NCIMB 10746 (NR118999) | 1199  1197  1196 | 99  100  100 | 0.0  0.0  0.0 | 99  98  98 |
| **AB973386 Strain R60 1103 nt, best scores**  *Clostridium argentinense* ATCC 27322 (NR029232)  *Clostridium subterminale* NCIMB 10746 (NR118999)  *Clostridium schirmacherense* AP15 (NR042448) | 880  879  875 | 96  96  96 | 0.0  0.0  0.0 | 81  81  81 |
| **AB973387 Strain R61 1064 nt, best scores**  *Clostridium argentinense* ATCC 27322 (NR029232)  *Acetoanaerobium noterae* NOT-3 (NR104848)  *Clostridium* *sticklandii* DSM 519 (NC102880) | 226  630  792 | 23  78  100 | 9e-82  9e-87  1e-85 | 88  74  73 |
| **AB973388 Strain R63 1094 nt, best scores**  *Acetoanaerobium noterae* NOT-3 (NR104848)  *Clostridium argentinense* ATCC 27322 (NR029232)  *Clostridium schirmacherense* AP15 (NR042448) | 223  276  223 | 22  29  29 | 7e-83  5e-84  2e-82 | 89  84  84 |
| **AB973389 Strain R90 1101 nt, best scores**  *Clostridium* *bifermentans* JCM 1386 (NR113323)  *Clostridium* *bifermentans* ATCC 638 (NR119066)  *Clostridium* *sordellii* JCM 3814 (NR113140) | 977  968  964 | 99  99  99 | 0.0  0.0  0.0 | 88  88  88 |
| **AB973390 Strain R91 1106 nt, best scores**  *Clostridium* *bifermentans* JCM 1386 (NR113323)  *Clostridium* *bifermentans* ATCC 638 (NR119066)  *Clostridium* *sordellii* JCM 3814 (NR113140) | 1078  1066  1049 | 100  100  98 | 0.0  0.0  0.0 | 97  96  97 |
| **AB973391 Strain R96 1059 nt, best scores**  *Clostridium* *bifermentans* JCM 1386 (NR113323)  *Clostridium* *sordellii* JCM 3814 (NR113140)  *Clostridium* *bifermentans* ATCC 638 (NR119066) | 1037  1025  1025 | 100  100  100 | 0.0  0.0  0.0 | 98  97  97 |
| **AB973392 Strain R97 1111 nt, best scores**  *Clostridium argentinense* ATCC 27322 (NR029232)  *Clostridium subterminale* NCIMB 10746 (NR118999)  *Clostridium schirmacherense* AP15 (NR042448) | 975  973  970 | 100  100  100 | 0.0  0.0  0.0 | 87  87  86 |
| **AB973393 Strain R107 1239 nt, best scores**  *Clostridium* *bifermentans* JCM 1386 (NR113323)  *Clostridium* *bifermentans* ATCC 638 (NR119066)  *Clostridium* *sordellii* JCM 3814 (NR113140) | 1233  1220  1207 | 100  100  100 | 0.0  0.0  0.0 | 99  98  97 |

*Number of 16S ribosomal RNA sequences (Bacteria and Archaea) in the GenBank sequence database.
